# Ivabradina na Síndrome Postural Ortostática Taquicardizante. É Tudo o que Precisamos?

**DOI:** 10.36660/abc.20250666

**Published:** 2026-03-13

**Authors:** Helena Nogueira Brasil, Bianca Lopes Cunha, Ana Taísa Barbosa de Mendonça, Francisca Tatiana Moreira Pereira, Eduardo Arrais Rocha

**Affiliations:** 1 Universidade Federal do Ceará (UFC-CE) Hospital Walter Cantídeo Fortaleza CE Brasil Hospital Walter Cantídeo, Universidade Federal do Ceará (UFC-CE), Fortaleza, CE – Brasil; 2 Centro de Ciências da Universidade Federal do Ceará (UFC-CE) Fortaleza CE Brasil Centro de Ciências da Universidade Federal do Ceará (UFC-CE), Fortaleza, CE – Brasil; 3 Universidade Federal do Ceará (UFC-CE) Faculdade de Medicina Fortaleza CE Brasil Faculdade de Medicina da Universidade Federal do Ceará (UFC-CE), Fortaleza, CE – Brasil; 4 Centro de Arritmia do Ceará Fortaleza CE Brasil Centro de Arritmia do Ceará, Fortaleza, CE – Brasil

**Keywords:** Síndrome da Taquicardia Postural Ortostática, Ivabradina, Intolerância Ortostática

A revisão sistemática "Uso da Ivabradina na Terapêutica de Pacientes Portadores da Síndrome da Taquicardia Postural Ortostática (POTS): Uma Revisão Sistemática", publicado na ABC Cardiol,^1^ fornece uma relevante discussão sobre as evidências do uso da ivabradina na terapêutica da Síndrome Postural Ortostática Taquicardizante (SPOT ou POTS), uma condição ainda subdiagnosticada e carente de tratamento farmacológico específico.^2^ A revisão analisa uma medicação já disponível no mercado, com perfil farmacológico bem conhecido e favorável e que apresenta, em geral, pouca repercussão hemodinâmica, uma vez que é capaz de reduzir a frequência cardíaca por atuar no nó sinusal, sem afetar a condução atrioventricular ou alterar a pressão arterial.^2,3^ Essa droga tem indicação já bem estabelecida na taquicardia sinusal inapropriada^3^ e em pacientes com insuficiência cardíaca congestiva com fração de ejeção reduzida, que mantém frequência cardíaca (FC) média elevada (>70 bpm) apesar do uso de betabloqueadores.^4^

Um ponto forte do trabalho foi reunir de forma clara e organizada as evidências disponíveis, destacando o potencial dessa medicação na redução da frequência cardíaca, melhora dos sintomas ortostáticos e ausência de efeitos adversos significativos na maioria dos casos.

Apesar da qualidade do trabalho, algumas limitações devem ser ressaltadas. A amostra total foi pequena e heterogênea, o que impede conclusões robustas e a ausência de metanálise limita a força da evidência. Os diferentes desenhos dos estudos (retrospectivos e prospectivos) e a falta de padronização na avaliação dos sintomas limitam a comparabilidade.

Estudos de fisiologia do professor Guyton^5^ na década de 60 já demonstravam o padrão de curva em sino da FC x débito cardíaco (DC), quando a partir de determinadas elevações na FC, ocorreriam reduções no DC (Figura1). Excessivas elevações na FC durante a ortostase, particularmente maiores que 120 bpm, reduzem o débito cardíaco independente do mecanismo fisiopatológico. Tais alterações são muito sensíveis a volemia do paciente, posição de ortostase com consequente redução no retorno venoso.^6^ Essas quedas no débito cardíaco durante taquicardias excessivas podem ocorrer na taquicardia sinusal inapropriada (TSI), na POTS e nas síndromes vasovagais.

**Figura 1:**
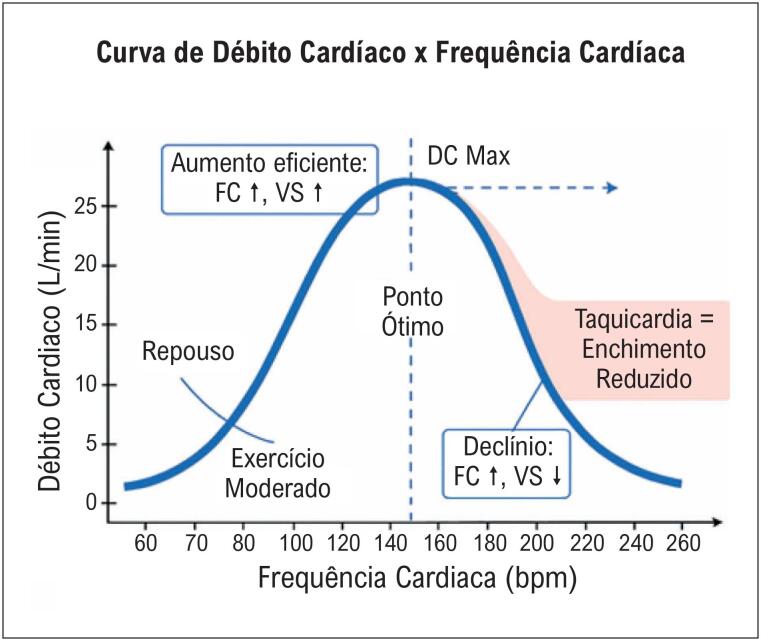
Curva em sino, demonstrando que o débito cardíaco cresce até um pico por volta de 120 bpm e depois cai de forma acentuada quando a frequência ultrapassa 160–180 bpm. Esse limite de compensação depende muito da volemia e do retorno venoso. Modificada de Sugimoto, Sagawa e Guyton.^5,6^

Conforme apresentado no trabalho, apenas no estudo de Taub et al.^7^ foi individualizado o perfil hemodinâmico dos pacientes com POTS, sendo todos os pacientes do perfil hiperadrenérgico, demonstrando um resultado superior aos outros estudos. É provável que a taquicardia sinusal em alguns padrões hemodinâmicos da POTS seja realmente inapropriada e não compensatória, justificando a melhora com esse fármaco em alguns subtipos.

Pacientes com POTS apresentam respostas hemodinâmicas heterogêneas, sendo a resposta hiperadrenérgica apenas uma delas. Oliveira et al.^8^ descreveram quatro respostas hemodinâmicas distintas no POTS durante o teste de inclinação. Esse estudo encontrou: 35% com queda da resistência vascular periférica (RVP) e manutenção ou elevação do volume sistólico (VS) (perfil neuropático); 37,5% com queda no VS + manutenção/elevação na RVP (perfil hipovolêmico); 17,5% com queda simultânea no VS e RVP (perfil misto); 10% com aumento de ambos VS e RVP (perfil hiperadrenérgico).

O padrão hiperadrenérgico é o fenótipo onde essa droga tem melhor perfil, pois atua seletivamente sobre o nó sinusal, controlando o excesso cronotrópico sem risco de agravar a vasoconstrição já presente.^8^

No fenótipo neuropático, onde há falha na vasoconstricção adequada durante a ortostase, a ivabradina pode reduzir a taquicardia compensatória, com risco de piorar sintomas por limitar o mecanismo cronotrópico que ajuda a manter o débito cardíaco diante da queda na resistência periférica.

De forma semelhante, no fenótipo hipovolêmico, a queda do volume sistólico é o gatilho principal, compensado por vasoconstrição periférica e aumento na FC. Nesse caso, a ivabradina pode ser benéfica para controlar o excesso de aumento na frequência, porém se usada isoladamente pode reduzir o débito cardíaco, já que o aumento de FC é um dos mecanismos compensatórios. Nesses casos, é prudente considerar seu uso após correção da hipovolemia (Tabela 1).

**Tabela 1:** Padrões hemodinâmicos de POTS e a resposta esperada ao uso de ivabradina

Mecanismo predominante	Descrição	Possível Uso da Ivabradina
**Fenótipo hiperadrenérgico** (Excessiva descarga simpática)	FC elevada, com manutenção/aumento do VS e RVP.	Favorável → reduz a FC sem impactar na PA, controlando a taquicardia "desproporcional".
**Fenótipo neuropático** (Déficit vasoconstritor)	Queda da RVP, com VS preservado.	Cauteloso → risco de prejudicar a compensação hemodinâmica.
**Fenótipo hipovolêmico** (Déficit de pré-carga).	Queda do VS, compensada por vasoconstrição (aumento da RVP).	Limitado → redução na FC pode prejudicar o DC. Deve-se corrigir a hipovolemia antes da medicação.
**Fenótipo misto** (Hipovolêmico + neuropático).	Queda simultânea do VS e da RVP.	Arriscado → taquicardia é compensatória; Risco de agravar a intolerância ortostática.

FC: frequência cardíaca; VS: volume sistólico; RVP: resistência vascular periférica; PA: pressão arterial; DC: débito cardíaco.

O fenótipo misto (hipovolêmico + neuropático) é o grupo mais grave e que apresentou maior aumento da FC.^8^ Há duplo comprometimento: baixa pré-carga e resposta vasomotora inadequada. A ivabradina poderá piorar a perfusão sistêmica, visto que o aumento da FC é um mecanismo compensatório crítico. Nesse subtipo, a reposição volêmica e vasoconstritores são a primeira linha de tratamento (Tabela 1).

Na TSI, uma meta-análise de nove estudos prospectivos com ivabradina incluiu 145 pacientes, dos quais ≥ 70% eram mulheres. Os estudos foram pequenos e não tiveram poder estatístico adequado. No entanto, todos apresentaram redução na FC máxima ou média em repouso, ou ambas, com melhora completa ou considerável dos sintomas com ivabradina.^9^

## Perspectivas Futuras

O cenário atual aponta para a ivabradina como uma alternativa promissora no manejo da POTS, especialmente do tipo hiperadrenérgico. Entretanto, são necessários ensaios clínicos randomizados, multicêntricos e com amostras mais amplas para demonstrar tal benefício em diversas populações. Estudos futuros deveriam explorar a estratificação fenotípica e o impacto na qualidade de vida e funcionalidade, além da segurança a longo prazo.

Este trabalho de revisão publicado por ABC-2025-0347 reafirma a ivabradina como uma estratégia terapêutica viável e segura para POTS, mas reforça também a necessidade de mais estudos na área. Trata-se de uma linha de pesquisa com grande potencial de impacto clínico e social, dado o sofrimento funcional imposto pela síndrome e a carência de opções terapêuticas específicas. É provável que a ivabradina seja primeira linha de tratamento na TSI, mas apenas uma alternativa para determinados fenótipos da POTS.
