# Supplementary material for: Ivabradina na Síndrome Postural Ortostática Taquicardizante. É Tudo o que Precisamos?
Source: Arq Bras Cardiol. 2026 Mar 13;122(11):e20250666. [Article in Portuguese] doi: 10.36660/abc.20250666 (PMC13155788; doi:10.36660/abc.20250666)
Supplement: Translation

# Translation: Ivabradine in Postural Orthostatic Tachycardia Syndrome. Is That All We Need?

**DOI:** 10.36660/abc.20250666i

**Authors:** Helena Nogueira Brasil, Bianca Lopes Cunha, Ana Taísa Barbosa de Mendonça, Francisca Tatiana Moreira Pereira, Eduardo Arrais Rocha

**Affiliations:** 1 Fortaleza CE Brazil Hospital Walter Cantídeo, Universidade Federal do Ceará (UFC-CE), Fortaleza, CE – Brazil; 2 Fortaleza CE Brazil Centro de Ciências da Universidade Federal do Ceará (UFC-CE), Fortaleza, CE – Brazil; 3 Fortaleza CE Brazil Faculdade de Medicina da Universidade Federal do Ceará (UFC-CE), Fortaleza, CE – Brazil; 4 Fortaleza CE Brazil Centro de Arritmia do Ceará, Fortaleza, CE – Brazil

**Keywords:** Postural Orthostatic Tachycardia Syndrome, Ivabradine, Orthostatic Intolerance

The systematic review "Use of Ivabradine in the Treatment of Patients with Postural Orthostatic Tachycardia Syndrome (POTS): A Systematic Review" published in ABC Cardiol^1^ provides a relevant discussion on the evidence for the use of ivabradine in the treatment of Postural Orthostatic Tachycardia Syndrome (POTS), a condition that is still underdiagnosed and lacking specific pharmacological treatment.^2^ The review analyzes a medication already available on the market, with a well-known and favorable pharmacological profile, and which generally presents little hemodynamic impact, since it is able to reduce heart rate by acting on the sinus node, without affecting atrioventricular conduction or altering blood pressure.^2,3^ This drug has a well-established indication in inappropriate sinus tachycardia^3^ and in patients with congestive heart failure with reduced ejection fraction, who maintain a high average heart rate (HR) (>70 bpm) despite the use of beta-blockers.^4^

A strong point of the study was to gather the available evidence in a clear and organized manner, highlighting the potential of this medication in reducing heart rate, improving orthostatic symptoms, and the absence of significant adverse effects in most cases.

Despite the quality of the work, some limitations should be highlighted. The total sample size was small and heterogeneous, which prevents robust conclusions, and the absence of meta-analysis limits the strength of the evidence. The different study designs (retrospective and prospective) and the lack of standardization in symptom assessment limit comparability.

Physiological studies by Professor Guyton^5^ in the 1960s already demonstrated the bell-shaped curve pattern of heart rate (HR) versus cardiac output (CO), where, from certain elevations in HR, reductions in CO would occur (Figure 1). Excessive elevations in HR during orthostasis, particularly greater than 120 bpm, reduce cardiac output regardless of the pathophysiological mechanism. Such alterations are very sensitive to the patient's volemia, orthostatic position, with consequent reduction in venous return.^6^ These drops in cardiac output during excessive tachycardias can occur in inappropriate sinus tachycardia (IST), POTS, and vasovagal syndromes.

**Figure 1:** Bell curve, demonstrating that cardiac output increases to a peak around 120 bpm and then drops sharply when the rate exceeds 160–180 bpm. This compensation limit depends heavily on blood volume and venous return. Modified from Guyton.^5,6^

As presented in the work, only in the study by Taub et al.^7^ was the hemodynamic profile of patients with POTS individualized, with all patients having a hyperadrenergic profile, demonstrating a superior result compared to other studies. Likely, sinus tachycardia in some hemodynamic patterns of POTS is actually inappropriate and non-compensatory, justifying the improvement with this drug in some subtypes.

Patients with POTS exhibit heterogeneous hemodynamic responses, with the hyperadrenergic response being only one of them. Oliveira et al. (2023)^8^ described four distinct hemodynamic responses in POTS during the tilt table test. This study found: 35% with a decrease in peripheral vascular resistance (PVR) and maintenance or increase in stroke volume (SV) (neuropathic profile); 37.5% with a decrease in SV + maintenance/elevation in PVR (hypovolemic profile); 17.5% with a simultaneous decrease in SV and PVR (mixed profile); 10% with an increase in both SV and PVR (hyperadrenergic profile).

The hyperadrenergic pattern is the phenotype where this drug has the best profile, as it acts selectively on the sinus node, controlling chronotropic excess without the risk of worsening the vasoconstriction already present.^8^

In the neuropathic phenotype, where there is a failure of adequate vasoconstriction during orthostasis, ivabradine may reduce compensatory tachycardia, with the risk of worsening symptoms by limiting the chronotropic mechanism that helps maintain cardiac output in the face of decreased peripheral resistance.

Similarly, in the hypovolemic phenotype, the drop in stroke volume is the main trigger, compensated by peripheral vasoconstriction and an increase in heart rate. In this case, ivabradine may be beneficial in controlling the excessive increase in heart rate, but if used alone, it may reduce cardiac output, since the increase in heart rate is one of the compensatory mechanisms. In these cases, it is prudent to consider its use after correction of hypovolemia (Table 1).

**Table 1:** Hemodynamic patterns of POTS and the expected response to the use of ivabradine

Predominant mechanism	Description	Possible Use of Ivabradine
**Hyperadrenergic phenotype** (Excessive sympathetic discharge)	Elevated HR, with maintenance/increase in SV and PVR.	Favorable → reduces heart rate without impacting blood pressure, controlling "disproportionate" tachycardia.
**Neuropathic phenotype** (Vasoconstrictor deficit)	PVR decrease, with SV preserved.	Caution → risk of impairing hemodynamic compensation.
**Hypovolemic phenotype** (Preload deficit).	A drop in SV is compensated for by vasoconstriction (increased PVR).	Limited → a reduction in heart rate may impair cardiac output. Hypovolemia should be corrected before medication.
Mixed phenotype (Hypovolemic + neuropathic).	Simultaneous drop in SV and PVR.	Risky → tachycardia is compensatory; Risk of worsening orthostatic intolerance.

SV: stroke volume; PVR: peripheral vascular resistance.

The mixed phenotype (hypovolemic + neuropathic) is the most severe group and showed the greatest increase in HR.^8^ There is a double impairment: low preload and inadequate vasomotor response. Ivabradine may worsen systemic perfusion, since the increase in HR is a critical compensatory mechanism. In this subtype, volume replacement and vasoconstrictors are the first-line treatment (Table 1).

In TSI, a meta-analysis of nine prospective studies with ivabradine included 145 patients, of whom ≥ 70% were women. The studies were small and lacked adequate statistical power. However, all showed a reduction in maximum or mean resting heart rate, or both, with complete or considerable improvement of symptoms with ivabradine.^9^

## Future Perspectives

The current scenario points to ivabradine as a promising alternative in the management of POTS, especially the hyperadrenergic type. However, randomized, multicenter clinical trials with larger samples are needed to demonstrate this benefit in diverse populations. Future studies should explore phenotypic stratification and the impact on quality of life and functionality, as well as long-term safety.

This review paper, published by ABC-2025-0347, reaffirms ivabradine as a viable and safe therapeutic strategy for POTS, but also reinforces the need for more studies in the area. This is a line of research with great potential for clinical and social impact, given the functional suffering imposed by the syndrome and the lack of specific therapeutic options. Ivabradine will likely be a first-line treatment for TSI, but only an alternative for certain POTS phenotypes.
